# The Potential Cost-Effectiveness and Equity Impacts of Restricting Television Advertising of Unhealthy Food and Beverages to Australian Children

**DOI:** 10.3390/nu10050622

**Published:** 2018-05-15

**Authors:** Vicki Brown, Jaithri Ananthapavan, Lennert Veerman, Gary Sacks, Anita Lal, Anna Peeters, Kathryn Backholer, Marjory Moodie

**Affiliations:** 1 Centre for Population Health Research, School of Health and Social Development, Global Obesity Centre (GLOBE), Deakin University, Geelong, VIC 3220, Australia; jaithri.ananthapavan@deakin.edu.au (J.A.); Gary.sacks@deakin.edu.au (G.S.); Anita.lal@deakin.edu.au (A.L.); anna.peeters@deakin.edu.au (A.P.); kathryn.backholer@deakin.edu.au (K.B.); marj.moodie@deakin.edu.au (M.M.); 2 Deakin Health Economics, Centre for Population Health Research, School of Health and Social Development, Deakin University, Geelong, VIC 3220, Australia; 3 Cancer Research Division, Cancer Council NSW, Woolloomooloo, Sydney, NSW 2011, Australia; lveerman@griffith.edu.au; 4 School of Medicine, Griffith University, Gold Coast, QLD 4222, Australia

**Keywords:** economic evaluation, cost-effectiveness, obesity, pediatric

## Abstract

Television (TV) advertising of food and beverages high in fat, sugar and salt (HFSS) influences food preferences and consumption. Children from lower socioeconomic position (SEP) have higher exposure to TV advertising due to more time spent watching TV. This paper sought to estimate the cost-effectiveness of legislation to restrict HFSS TV advertising until 9:30 pm, and to examine how health benefits and healthcare cost-savings differ by SEP. Cost-effectiveness modelling was undertaken (i) at the population level, and (ii) by area-level SEP. A multi-state multiple-cohort lifetable model was used to estimate obesity-related health outcomes and healthcare cost-savings over the lifetime of the 2010 Australian population. Incremental cost-effectiveness ratios (ICERs) were reported, with assumptions tested through sensitivity analyses. An intervention restricting HFSS TV advertising would cost AUD5.9M (95% UI AUD5.8M–AUD7M), resulting in modelled reductions in energy intake (mean 115 kJ/day) and body mass index (BMI) (mean 0.352 kg/m^2^). The intervention is likely to be cost-saving, with 1.4 times higher total cost-savings and 1.5 times higher health benefits in the most disadvantaged socioeconomic group (17,512 HALYs saved (95% UI 10,372–25,155); total cost-savings AUD126.3M (95% UI AUD58.7M–196.9M) over the lifetime) compared to the least disadvantaged socioeconomic group (11,321 HALYs saved (95% UI 6812–15,679); total cost-savings AUD90.9M (95% UI AUD44.3M–136.3M)). Legislation to restrict HFSS TV advertising is likely to be cost-effective, with greater health benefits and healthcare cost-savings for children with low SEP.

## 1. Introduction

Childhood obesity is a significant public health issue worldwide [1]. Obesity in childhood is a risk factor for overweight and obesity in adulthood [2], resulting in both short-term and long-term negative health effects and highlighting the importance of obesity prevention for children and adolescents. Childhood and adolescent overweight and obesity also have a socioeconomic gradient. Youth with a low socioeconomic position (SEP) are at a greater risk of overweight and obesity compared to youth with a higher SEP [3]. Population obesity prevention interventions that reduce these inequities are increasingly being recognised as a critical component of successful obesity prevention efforts [4].

A diet high in fat, salt and sugar (HFSS) is a key modifiable risk factor for childhood obesity and diet-related non-communicable diseases. In 2010, the World Health Organization (WHO) concluded that television (TV) advertising influences children’s food preferences, purchase requests and consumption patterns and published a set of recommendations on food marketing to children (of which TV advertising is one component) [5].

Evidence also suggests there is a socioeconomic gradient in TV viewing patterns [6,7,8]. Australian children with a lower SEP are more likely to watch TV and for longer periods of time compared to those with a higher SEP [6,7]. This means that children with a lower SEP are likely to be exposed to greater levels of HFSS TV advertising compared to children with a higher SEP. Restricting HFSS TV advertising therefore has the potential to not only reduce obesity prevalence among youth, but also reduce socioeconomic inequities in obesity prevalence.

Several countries have introduced regulatory schemes to reduce or restrict HFSS advertising. Statutory regulations have resulted in bans on TV advertising to children in countries or jurisdictions such as Quebec, Sweden and Norway [9]. Commercial broadcasters in Australia must meet legislated broadcasting standards, and the Australian Communications and Media Authority’s (ACMA) Children Television Standards (CTS) currently restrict the broadcasting of advertising during so-called ‘P’ (pre-school) programs [10]. The food industry introduced two self-regulatory, voluntary codes aimed at reducing exposure of children to marketing, managed by the Australian Food and Grocery Council (AFGC) [11].

The effectiveness of combinations of government and self-regulation in reducing the exposure of children to HFSS TV advertising is however limited [12]. Critics of Australia’s self-regulated schemes note the relatively weak commitments that form the initiatives, along with the lack of sanctions for breaches of the codes [13]. Much of the TV content that Australian children aged 0–14 years watched in 2016 was not specifically categorised as children’s content, with 20% of the top 30 programs classified as reality programs, 7% light entertainment and 20% movies [14]. A report by the Australian National Preventive Health Agency (ANPHA) in 2012 [15] found that while advertising of non-core foods during children’s classified (‘C’) programs is low, rates of HFSS advertising during children’s peak free-to-air (FTA) viewing times is up to 6.5 advertisements per hour. Many Australian children are therefore exposed to advertising of HFSS foods whilst watching shows not specifically classified for children.

Despite the strong program logic that links exposure to HFSS TV advertising to increased HFSS consumption and obesity, establishment of the effectiveness of its restriction is challenging [16]. Real world evidence from jurisdictions that have implemented bans on HFSS advertising has shown promising results on intermediate outcomes, including a reduction in expenditure on HFSS foods following a ban on TV advertising in Quebec [17] and a reduction in HFSS drink expenditure following a move to self-regulation and co-regulation in the UK [18]. Rigorous estimation of effectiveness from an obesity prevention perspective is however complicated by the environmental-type nature of the intervention, the potential role of confounding factors and the time lag between the change in exposure and change in body mass index (BMI) being longer than many study timeframes. A relatively small number of studies have quantified the obesity-related effects of reducing children’s exposure to HFSS TV advertising using burden of disease or cost-effectiveness approaches and “best available” evidence [19,20,21,22,23]. The key limitation of these modelling studies has been the reliance on individual studies for the estimation of effect of TV advertising restrictions on BMI [24,25,26,27]. This has resulted in the modelled effect size varying, from −0.03 kg/m^2^ [23] to −1 kg/m^2^ [19,20] (Supplementary Information 1).

This paper aims to strengthen the evidence base on the potential cost-effectiveness of legislation to restrict HFSS TV advertising from an obesity prevention perspective, by (i) undertaking analyses using a synthesis of the evidence; (ii) accounting for differences in benefits and costs by SEP; and (iii) exploring thresholds for minimum BMI effect required to achieve cost-effectiveness. This will provide important information on the potential population level impact of the intervention, plus new information on the intervention’s potential to reduce health inequities across socioeconomic groups.

## 2. Materials and Methods

### 2.1. Current Practice

Australian legislation currently prohibits TV advertising during ‘P’ classified programs [10]. TV networks must screen at least 260 h of Children’s (‘C’) programs per year in ‘C’ time bands (7 am–8:30 am and 4 pm–8:30 pm weekdays, 7 am–8:30 pm weekends and school holidays). ‘C’ programs must not contain advertisements of more than 5 min in total per 30 min and content must not mislead or deceive children, put undue pressure on purchases or contain promotions by popular children’s characters immediately before, during or after a ‘C’ or ‘P’ period [10].

### 2.2. The Intervention

The proposed intervention was defined as legislation to implement time-based restrictions of unhealthy food and beverage marketing to children under 16 years of age on FTA TV until 9:30 pm [28,29]. Peak TV viewing periods for Australian children are from 8 am–9 am and 7 pm–8 pm [30], and therefore legislation would reduce exposure to HFSS advertising. It should be noted that older adolescents and adults may also benefit from reduced exposure to HFSS advertising, however modelling has been undertaken in line with current policy recommendations (focusing on benefits for children under 16 years) [26,27]. Whilst children aged under five years may also be exposed to less HFSS TV advertising as a result of the intervention, this was considered less likely given their relatively young age and higher likelihood of watching ‘P’ programs. The intervention effect estimation was therefore limited to children aged between five and 15 years. We assumed that baseline viewing in the intervention population occurs during the time period of the proposed intervention on FTA TV, and tested assumptions around viewing patterns on FTA vs. non-FTA TV in sensitivity analyses.

### 2.3. Assessment of Benefit

A scoping review of the literature was conducted to inform the evidence of effect. Searches were conducted by one author (VB), using the Scopus and EBSCOHost academic databases. “Gold standard” evidence to inform modelling would consist of BMI effect estimates from reduced exposure to HFSS TV advertising from high-quality randomised controlled trials (RCTs) conducted in natural settings [19,23]. A search was conducted for such evidence (Supplementary Information 2), with no relevant studies identified in the literature.

**Figure 1:**
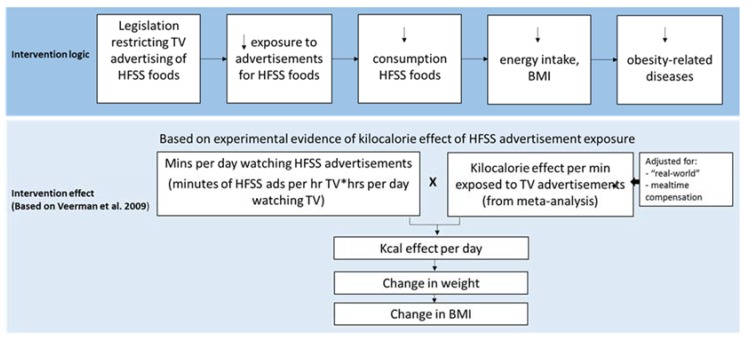
Logic pathway for modelling the effect of the intervention. BMI = body mass index. HFSS = high in fat, salt and sugar. Hrs = hours. Kcal = kilocalorie. Mins = minutes. TV = television.

Because of this absence of “gold standard” evidence, we defined the assessment of benefit of the intervention using a method previously used [19], whereby the effect is estimated based on the relationship between food promotion and consumption behaviours [16,31,32,33]. The logic pathway of the intervention effect is given (Figure 1).

A scoping search was conducted for reviews and systematic reviews reporting the effects of TV advertising on consumption in children and adolescents (Supplementary Information 2). Results of keyword searches were reviewed, with the meta-analysis by Boyland et al. [33] identified due to its immediate relevance and most recent publication date (2016). Boyland et al. [33] reviewed the evidence for an association between exposure to HFSS food advertising and energy intake and reported mean differences between exposure and control conditions. In order to model the effect of the intervention to BMI, estimates of a change in kilocalorie (kcal) (energy intake) per minute exposed was required. We conducted a meta-analysis of all studies in the review by Boyland et al. [33] that reported results for children that could be converted to kcal effect per minute exposed to TV advertisements using a random effects model. Heterogeneity was explored using Cochran’s Q and *I*^2^. Full meta-analysis methods and the characteristics of included studies are reported in Supplementary Information 3.

Studies included in the meta-analysis reported effects from acute exposure in controlled settings. Consumption under experimental conditions may not necessarily reflect consumption in natural settings, due to factors such as scrutiny, context and differences in time horizons and choice sets [34]. However, limited evidence on the external validity of such study findings exists. We therefore applied a crude adjustment factor for translation of experimental effects to the real-world, assuming that 50% of the effect would be maintained under non-experimental conditions (Table 1). Compensatory intake effect at mealtimes was also included, given evidence suggesting that children may compensate for snacks between meals by consuming less at mealtimes [35]. We therefore reduced the effect estimate, to conservatively estimate overall impact of exposure to HFSS TV advertisements on energy intake in children by assuming increased energy intake was a product of snacking between meals (Table 1). The derived estimate of effect was multiplied by estimates of time per day spent watching HFSS TV ads by age and SEP (Table 1, Supplementary Information 4). The change in mean energy intake as a result of the intervention was then converted to change in mean BMI using the validated equations by Hall et al. [36], with assumptions tested in sensitivity analyses.

### 2.4. Assessment of Costs

Fixed costs were estimated as the cost of passing legislation [37] (Table 1). Ongoing costs comprised the salary costs of two administration and compliance officers [21], including salary on-costs [38,39,40]. Potential loss of revenue to TV networks resulting from reduced advertising was tested in sensitivity analysis. From a societal perspective, the base case assumed that potential loss of revenue for the food and advertising industries would be compensated by revenue for the advertisement of other products or consumer expenditure on other goods and services. Data on healthcare costs were obtained from the Australian Institute of Health and Welfare (AIHW) for 2001 [41], and inflated to 2010 Australian dollar prices using the Health Price Index [42].

### 2.5. Cost-Effectiveness Modelling

Cost-effectiveness modelling was undertaken from a limited societal perspective, with the time horizon for estimating costs, cost-savings and health benefits being rest-of-life or 100 years for the 2010 Australian population. Costs, cost-savings and health benefits were discounted at 3% and were presented as 2010 values.

Modelling was undertaken as (i) a whole population analysis, and (ii) an analysis by area-level SEP, using the Socio-Economic Indexes for Areas (SEIFA) IRSD quintiles 1 (most disadvantaged) and 5 (least disadvantaged) [43]. A proportional multi-state, multiple cohort life table model was used to estimate obesity-related health outcomes and healthcare cost-savings. The model used data from the Australian Health Survey 2011–2012 [44] and disease epidemiology from the Global Burden of Disease study [45]. Modelling by SEP group incorporated SEIFA quintile specific data, including disease incidence, mortality rate, BMI distribution and population number [37]. Potential impact fractions estimated the proportional reduction in disease incidence that would occur through reduced exposure to a risk factor as a result of an intervention. Disease-specific life tables estimated mortality and morbidity for nine obesity-related diseases (ischaemic heart disease, hypertensive heart disease, ischemic stroke, diabetes, colorectal cancer, kidney cancer, breast cancer, endometrial cancer and osteoarthritis). Interventions were compared against a “no intervention” comparator, where the distribution of BMI in the 2010 reference year Australian population remained unchanged [46].

All modelling was undertaken in Microsoft Excel 2016. Uncertainty analysis around key input parameters was estimated using Monte Carlo simulation (2000 iterations) using the Excel add-in Ersatz (version 1.35) [47] to estimate 95% uncertainty intervals (95% UI). Quality of life in children was incorporated using values from the literature [48]. Cohort-based modelling allowed disease-related benefits which are not present in childhood to be estimated due to lingering BMI effects. It was assumed that the BMI effect as a result of the intervention at age 15 years was maintained into adulthood, allowing the estimation of health benefits and healthcare cost-savings over the lifetime. Incremental cost-effectiveness ratios (ICERs) were calculated by dividing the difference in the net cost of the intervention by the difference in the net health benefit. ICER results were presented on a cost-effectiveness plane. The AUD50,000 per health adjusted life year (HALY) threshold was used to determine cost-effectiveness, as per Australian benchmarks [49].

### 2.6. Sensitivity Analyses

The base case assumes no loss of advertising revenue for broadcasters, given the likelihood of new advertising contracts to fill existing advertising periods. The effect of this assumption was tested in sensitivity analyses, assuming a short-term (one year) loss of revenue before networks recoup these costs through new advertising contracts (Table 1) (Supplementary Information 5).

Multi-variate sensitivity analysis was undertaken by varying key input parameters in a “worst-case” analysis (Table 1). Worst-case analysis incorporated network loss of revenue, a smaller effect estimate derived from the meta-analysis (Supplementary Information 5) and greater adjustment for translation of effect from experimental to real-world settings. Worst-case analysis also varied the assumption that all TV viewing occurred on FTA TV. Pay TV and streamed services have gained popularity amongst Australian TV viewing audiences, although limited data exists on the proportion of children’s viewing time using these services. A recent report by Deloitte Australia [54] cited that 22% of total viewing time is now via paid or streamed services, although this was based on a non-representative survey of people aged over 14 years. In lieu of more rigorous data, the worst-case analysis adjusted the mean minutes spent watching TV per day to assume that 22% of total viewing time was on streamed services with no exposure to HFSS advertising (for example, services like Netflix).

### 2.7. Threshold Analysis

Given the relative uncertainty around the evidence of effect, a threshold analysis was undertaken to determine the minimum effect size required to return a mean ICER less than the AUD50,000 per HALY threshold [49]. Analysis was undertaken using base case intervention costs, and additionally incorporating loss of short-term revenue to TV networks (Table 1).

### 2.8. Implementation Considerations

The broader impacts of an intervention that may not necessarily be captured within an economic evaluation should also be considered in order to take into account the wide range of factors important to decision-makers when setting priorities [55]. Cost-effectiveness results are discussed alongside implementation considerations, which qualitatively assess the strength of evidence, feasibility, acceptability and sustainability of the proposed intervention.

## 3. Results

Results suggest that an intervention restricting TV advertising of HFSS food and beverages to children would cost AUD5.9M (95% UI AUD5.8M–AUD7M). The intervention would result in a small mean decrease in energy intake (approximately 115 kJ/day) and a small mean BMI reduction (0.352 kg/m^2^) at the population level (Table 2). The cost-effectiveness modelling showed that the intervention would be dominant (i.e., cost-saving and health promoting), resulting in 88,396 HALYs saved (95% UI 54,559–123,199) and total cost-savings of AUD777.9M (95% UI AUD369.8M–AUD1.2B) at the population level over the lifetime (Table 2).

**Figure 2:**
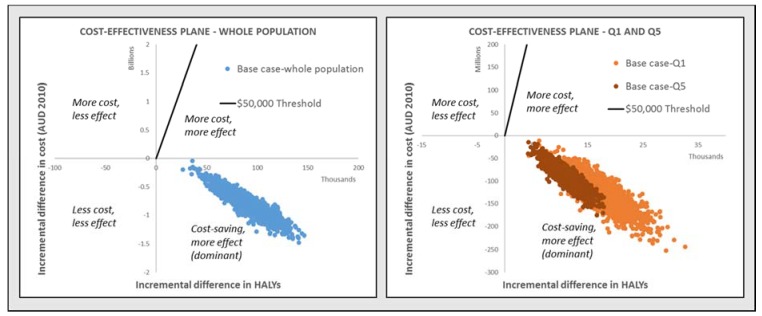
Cost-effectiveness planes, intervention restricting TV advertising of HFSS food and beverages to Australian children aged 5–15 years.

Importantly, the intervention may reduce health inequities, resulting in 1.5 times more HALYs and 1.4 times higher total cost-savings in children living in the most disadvantaged areas (Q1) compared to the least disadvantaged areas (Q5) (Table 2). The cost-effectiveness planes in Figure 2 demonstrate the dominance of the intervention, at both the population level and for the most disadvantaged (Q1) and least disadvantaged (Q5) quintiles.

The intervention would also be dominant when short-term loss of revenue to TV networks is included (probability of: cost-effectiveness 100%, dominance 99.9%) (Supplementary Information 5). Worst-case sensitivity analysis results also suggested that the intervention would remain dominant when varying input parameters to result in a much smaller effect estimate (population level mean BMI reduction 0.13 kg/m^2^, Supplementary Information 5), combined with a short-term loss of revenue to TV networks (probability of: cost-effectiveness 99.5%, dominance 83.5%).

Threshold analysis demonstrated that the effect size (expressed as the relationship between ‘minutes of exposure to TV ads for HFSS food’ and ‘energy intake’) could be reduced by more than two orders of magnitude (from a lower range estimate of 15.5 kcal per minute exposed, to <0.1 kcal per minute exposed) for the intervention to remain cost-effective. In this scenario, assuming base case intervention cost, a BMI reduction of 0.0004 kg/m^2^ would result in a mean ICER under the AUD50,000 cost-effectiveness threshold (mean ICER AUD44,688 per HALY saved (95% UI AUD28,815–79,516)). Under the base case scenario for intervention benefits, short-term loss of revenue to TV networks could be more than seven times higher (~$700 million), and the intervention would remain dominant (mean ICER per HALY saved 95% UI dominant-AUD7215).

## 4. Discussion

An intervention restricting the exposure of Australian children to TV advertising of HFSS food and beverages is likely to be cost-saving over the lifetime of the cohort under the base case and all sensitivity analyses modelled, with greatest health benefits and cost-savings accrued by children living in the most disadvantaged areas (Q1) compared to children living in the least disadvantaged areas (Q5). To the best of our knowledge, this is the first time that an intervention restricting HFSS TV advertising to children has been modelled taking into account differences in advertising exposure by SEP.

Results suggest the significant potential of legislation to restrict HFSS TV advertising to children in addressing health inequities, in a cost-effective manner. Given that the differences in effect estimates by SEP were determined by differences in TV viewing time within our analysis, the differences in health benefits and total cost-savings by measure of area-level disadvantage may actually be under-estimates. However, if the content of what children are watching on TV also differs by SEP (for instance, FTA vs. paid/streamed services with varying levels of advertisement exposure), the differences in health benefits and total cost-savings between children with low SEP and children with high SEP may vary in other ways. More evidence quantifying the differential level and type of exposure of HFSS advertising to children by SEP is required to better inform this analysis. Further, this study only examined differences between SEP quintile one and five. Analysis across the entire socioeconomic spectrum is required, in order to gain a complete understanding of the equity-related effects of legislation to restrict HFSS TV advertising across all socioeconomic groups.

The most significant challenge to date remains the availability of rigorous evidence of effect of the intervention to better inform analysis (Table 3). While this study is based on strong program logic and the best available evidence of effect size, there is currently only limited evidence from real-world implementations of advertising restrictions. As more jurisdictions implement advertising restrictions (e.g., Chile, Canada), this will present increased opportunities to evaluate population-level impacts. Results from our threshold analyses demonstrate the small effect size (compared to our modelled effect estimates and meta-analyses based on previously published studies) required for the intervention to be considered cost-effective, further supporting the logic for further intervention in this area. Legislation restricting the exposure of children to TV advertising of HFSS foods and beverages is being implemented internationally (Table 3). Ireland has recently announced a comprehensive ban with a push for a 9 pm watershed on advertising of HFSS products on TV and radio, along with regulation across other advertising mediums. Whilst the Code of Practice has not yet been released by the Irish Department of Health, this demonstrates the feasibility of the intervention given the necessary political will.

Our cost-effectiveness results are in keeping with previously published findings in Australia [21], The United States [23] and internationally [22], that interventions to restrict TV advertising of HFSS food and beverages are considered cost-effective (Supplementary Information 1). Whilst results are not directly comparable due to methodological differences, there is now a growing body of evidence that the societal benefits of an intervention restricting the exposure of children to HFSS TV advertising outweigh the potential costs of legislation. In addition, our paper provides new evidence on the potential for cost-effectiveness of the intervention when incorporating what we consider to be realistic short-term loss of revenue to TV networks. In 2007, ACMA cited concerns regarding the impact of restricting food and beverage advertising to children on the production of children’s TV programs by networks [57]. The inclusion of network costs, whilst relatively uncertain given the commercial sensitivity of trying to estimate loss of revenue for a hypothetical intervention, goes some way towards addressing this issue. Our analyses including loss of advertising revenue to networks demonstrates that the intervention remains cost-effective even when incorporating these costs (Supplementary Information 5), suggesting the potential for cost-effective government reimbursement if required.

By limiting the intervention population to children aged five to 15 years, we may have under-estimated the cost-effectiveness of the intervention at the population level. Studies have suggested that HFSS TV advertising may also have an effect on food-related behaviours and attitudes in older adolescents and adults, however at this time the evidence is inconclusive [33] and therefore we have not included potential health benefits and cost-savings accruing to older adolescents and adults as a result of the intervention into our analyses. Whilst this likely under-estimates cost-effectiveness, more evidence is required in order to capture the full public health benefits of legislation to reduce exposure to HFSS TV advertising across the entire child and adult population.

Limitations of our study include the assumption of maintenance of BMI effect into adulthood and the modelled effect estimate used for base case and sensitivity analyses, taken from meta-analysis of a small number of studies conducted in highly controlled experimental conditions. We have tried to compensate for this suspected bias by adjusting the effect estimate for a more plausible translation to a real-world setting, and for compensatory meal behaviours of children who snack. Adjustments were made using best-available evidence and logic pathways, however a more rigorous understanding of the effects of TV advertising on the consumption of HFSS food and beverages in children is clearly an area for significant future research.

It is likely that the food industry would oppose the introduction of more stringent TV advertising legislation, and this is recognised as a potential barrier to the implementation of this public health policy [58]. Whilst our analysis took a limited societal perspective, we have not included loss of revenue to the food industry as a result of the intervention. We have instead assumed that any loss of food industry revenue from a reduction in demand for HFSS food and beverages would be compensated in increased demand for non-discretionary foods or other items, and therefore doesn’t result in a loss to society. However, this is yet to be rigorously and definitively tested. In addition to its effect on consumption, advertising is also designed to influence market share within a category, and the impact of advertising restrictions on individual companies is not analysed in this paper.

Finally, television advertising is only one medium used for marketing of HFSS food and beverages, and although traditional TV viewing is still the most dominant promotional channel, it may be waning [16]. Changes to technology have led to the development and implementation of comprehensive approaches to marketing focused on brand and relationship development, including advertising across mediums, sponsorship, product placement, brand mascots and celebrity endorsement, sales promotion, labelling and point-of-purchase displays [5]. This analysis did not take into account potential shifts in advertising from TV to other media in response to the intervention. Ideally this intervention would be combined with more comprehensive marketing restrictions across multiple forms of media, as well as form part of a broader obesity prevention strategy across health and non-health sectors (for example, fiscal, built environment, education interventions) [28,29]. Results from this study form part of a broader body of work into obesity prevention priority-setting in Australia, using standardised methods [37,46,59] and designed to better inform obesity prevention policy. Other interventions found to be cost-saving to date include sugar-sweetened beverage (SSB) taxation [37] and reformulation or package size caps of SSBs [46].

## 5. Conclusions

This study indicates the significant potential of legislation restricting TV advertising of HFSS food and beverages to Australian children as a cost-effective intervention, with greatest benefits for the most socioeconomically disadvantaged. All modelled scenarios and sensitivity analyses resulted in dominance (i.e., an intervention that is both cost-saving and improves long term health outcomes). Our analysis by measure of area-level socioeconomic disadvantage demonstrates greater health benefits and total cost-savings in those living in the most disadvantaged socioeconomic areas compared to those living in the least disadvantaged areas. Furthermore, results from our threshold analysis demonstrate the very small population level effect size required for the intervention to be considered cost-effective.

## Figures and Tables

**Table 1:** Key model variables.

Parameters	Mean Values and 95% UI	Data Source and Assumptions
*Intervention effect estimate*		
Mean minutes per day watching TV, by age and SEIFA IRSD quintile	See Supplementary Information 3	Sampled from a normal distribution, from Government sources [44]. Adjusted for time spent using TV screens for other uses [50].
Number of advertisements per hour for HFSS foods during children’s peak viewing times	3.4 (95% UI 1.9–5.2)	Sampled from a pert distribution, minimum 1.5 maximum 6.5 from a 2012 Australian review of outcomes for studies that reported non-core TV advertising during children’s peak viewing times (based on television audience patterns, generally weekday evenings and weekend mornings) [15]. Most likely 3.1 taken from Australian study 2017 [13].
TV advertisement length (seconds)	29.9 (95% UI 19.2–40.9)	Sampled from pert distribution, minimum 15, most likely 30, maximum 45. Based on logical reasoning and published estimates [20].
Reduction factor for application of experimental effect to real-world setting	0.50 (95% UI 0.16–0.85)	Sampled from a pert distribution, minimum 0.00, most likely 0.50, maximum 1.00. Based on assumption.
Mealtime compensation effect for snacking	0.37 (95% UI 0.22–0.61)	Sampled from a pert distribution, minimum 0.20, most likely 0.30, maximum 0.80 compensation index [35].
Kcal effect per minute of TV ad exposure per day	38 (95% UI 15.5–60.6)	Sampled from a normal distribution (mean 37.94, 95% UI 15.6–60.3), see Supplementary Information 2. After base-case reduction factor for application of experimental effect to real-world setting and mealtime compensation are applied, the kcal effect per minute of TV ad exposure per day is estimated as 12 (95% UI 3–27).
*Intervention cost estimate*		
Cost of legislation (including RIS process)	AUD1,089,650 (95% UI AUD940,351–1,240,624)	Sampled from a gamma distribution [37].
Weekly wage of personnel for legislation administration	AUD1242 (95% UI AUD1127–1358)	Sampled from a gamma distribution (mean 1240.90, se 58.90) Administrative and Support Services, fulltime adult [38].
Labour on-costs, 14% salary cost	AUD174 (95% UI AUD155–195)	Sampled from a pert distribution (+/−10%), from Government sources [39].
Annual leave loading, 17.5% weekly salary cost, 4 weeks per annum	AUD870 (95% UI AUD773–975)	Sampled from a pert distribution (+/−10%), from Government sources [40].
*Sensitivity analysis, worst case analysis*		
Assumed loss of network revenue, year one of intervention	2.5% (95% UI 0.4–5.1)	Sampled from a pert distribution (minimum 0, most likely 2%, maximum 7%), based on 2010 network advertising revenue of AUD3.9B [51,52,53].
Kcal effect per minute of TV ad exposure per day	27.6 (95% UI 19.3–35.8)	Sampled from a normal distribution (mean 27.6, 95% UI 19.5–35.7), see Supplementary Information 2.
Reduction factor for application of experimental effect to real-world setting	0.67 (95% UI 0.30–0.95)	Sampled from a pert distribution, minimum 0.00, most likely 0.75, maximum 1.00. Based on assumption.
Proportion of time spent watching paid or streamed TV services (assumed no advertisements)	0.22 (95% UI 0.20–0.24)	Sampled from a pert distribution, minimum 0.2, most likely 0.22, maximum 0.24 (+/−10%) from published estimate [54].

95% UI = 95% uncertainty interval; ABS = Australian Bureau of Statistics; AUD = Australian dollars; B = billion; BMI = body mass index; Kcal = kilocalories; RIS = regulatory impact statement; se = standard error; SEIFA = Socioeconomic Indexes for Areas Index of Relative Socioeconomic Disadvantage; TV = television.

**Table 2:** Cost-effectiveness results of restricting HFSS TV advertising.

Results	Children (5–15 Years)	Children Q1 (Most Disadvantaged)	Children Q5 (Least Disadvantaged)
Mean modelled kJ effect per day, children aged five to 15 years	−115 kJ/day	−132 kJ/day	−97 kJ/day
Mean modelled BMI effect, children aged five to 15 years	−0.352 kg/m^2^	−0.395 kg/m^2^	−0.299 kg/m^2^
Mean BMI effect maintained in adulthood	−0.345 kg/m^2^	−0.313 kg/m^2^	−0.282 kg/m^2^
Total HALYS saved over lifetime	88,396 (95% UI 54,559–123,199)	17,512 (95% UI 10,372–25,155)	11,321 (95% UI 6812–15,679)
Total healthcare cost-savings over lifetime	AUD783.8M (95% UI AUD375.6M–1.2B)	AUD127.5M (95% UI AUD59.8M–198.1M)	AUD92.1M (95% UI AUD45.4M–137.5M)
Total intervention costs	AUD5.9M (95% UI AUD5.8M–7M)	AUD1.2M ^#^ (95% UI AUD1.1M–1.3M)	AUD1.2M ^#^ (95% UI AUD1.1M–1.3M)
Total net cost-savings	AUD777.9M (95% UI AUD369.8–1.2B)	AUD126.3M (95% UI AUD58.7M–196.9M)	AUD90.9M (95% UI AUD44.3M–136.3M)
Net cost per HALY saved (ICER)	Dominant *	Dominant *	Dominant *
Probability of dominance	100%	100%	100%
Probability of cost-effectiveness	100%	100%	100%

^#^ Assumed attribution of one-fifth of total intervention cost to each quintile; * Dominant interventions result in health gains and cost-savings; 95% UI = 95% uncertainty interval based on 2000 simulations; AUD = Australian dollars; BMI = body mass index; HALYs = Health adjusted life years; ICER = Incremental cost-effectiveness ratio; kJ = kilojoule; 1 kilocalorie is equal to 4.184 kilojoules; M = million; Q = SEIFA IRSD quintile.

**Table 3:** Implementation considerations, intervention to restrict HFSS TV advertising to children.

Implementation Consideration:	Adjustments/Considerations	Overall Rating
**Strength of evidence**	Direct evidence of BMI effect of TV advertising of food and beverages HFSS from RCTs is currently not available. The intervention is modelled using an effect estimate derived from meta-analysis of non-naturalistic experimental evidence.	Low
**Acceptability**	*Food, media industry acceptability* Likely to be low. Marketing and advertising drives sales.	Low
	*Political acceptability* To date, political motivation to enact legislation has been low but may vary by political party and over time. International experience in countries such as Ireland and the United Kingdom suggests the potential for political acceptability.	Low
	*Consumer acceptability* Public support for government regulation of advertising of HFSS food and beverages to children is high [56].	High
**Feasibility**	This legislative intervention is feasible to implement in the Australian setting.	High
**Sustainability**	The intervention is sustainable once implemented. The ACMA already has regulatory responsibilities and can oversee the regulation of TV HFSS advertising. The sustainability of potential BMI effect is unknown, and more evidence is required on the effects of TV advertising of HFSS food and beverages in adults.	High
**Equity**	Children with low SEP may have more exposure to HFSS TV advertising than children with high SEP, due to differences in TV viewing practices.	Positive
**Side effects**	*Positive side effects* The intervention may have an impact on the food preferences and consumption behaviours of older children and adults.	Positive
	*Negative side effects* The intervention may result in loss of revenue to TV networks (likely to be short-term effect). The intervention may result in loss of revenue to food companies (although over the longer term it may be expected that companies adapt to market conditions).	
***Policy conclusion**:* The intervention demonstrates significant potential for cost-effectiveness, positive equity effects and is feasible, sustainable and acceptable to the Australian general public.		

ACMA = Australian Communications and Media Authority; BMI = body mass index; HFSS = High in fat, sugar or salt; PA = physical activity; RCT = randomised controlled trial; SEP = socioeconomic position; TV = television.

## Supplementary Materials

The following are available online at http://www.mdpi.com/2072-6643/10/5/622/s1, Supplementary Information 1: Summary of Published Findings on the Health Benefits of Restricting TV Advertising of HFSS Food and Beverages to Children, Supplementary Information 2: Scoping Search Strategy, Supplementary Information 3: Estimate of Effect, Meta-Analysis Results, Supplementary Information 4: Mean Minutes Spent Watching TV per Day, by Age and Quintile, Supplementary Information 5: Sensitivity Analysis Results.

## References

[1] World Health Organisation (2016). Report of the Commission on Ending Childhood Obesity.

[2] Stettler N., Iotova V. (2010). Early growth patterns and long-term obesity risk. Curr. Opin. Clin. Nutr. Metab. Care.

[3] Gebremariam M., Lien N., Nianogo R., Arah O. (2017). Mediators of socioeconomic differences in adiposity among youth: A systematic review. Obes. Rev..

[4] Peeters A., Backholer K. (2015). Reducing socioeconomic inequalities in obesity: The role of population prevention. Lancet Diabetes Endocrinol..

[5] World Health Organisation (2010). Set of Recommendations on the Marketing of Foods and Non-Alcoholic Beverages to Children.

[6] Bittman M., Sipthorp M. (2011). Turned on, Tuned in or Dropped Out? Young Children’s Use of Television and Transmission of Social Advantage.

[7] Mullan K. (2013). Growing Up in Australia: The Longitudinal Study of Australian Children Annual Statistical Report 2013.

[8] Cameron A.J., Spence A.C., Laws R., Hesketh K.D., Lioret S., Campbell K.J. (2015). A Review of the Relationship between Socioeconomic Position and the Early-Life Predictors of Obesity. Curr. Obes. Rep..

[9] Chambers S.A., Freeman R., Anderson A.S., MacGillivray S. (2015). Reducing the volume, exposure and negative impacts of advertising for foods high in fat, sugar and salt to children: A systematic review of the evidence from statutory and self-regulatory actions and educational measures. Prev. Med..

[10] Australian Government (2009). Children’s Television Standards 2009.

[11] Australian Food & Grocery Council Advertising to Children: AFGC; n.d. http://www.afgc.org.au/our-expertise/health-nutrition-and-scientific-affairs/advertising-to-children/.

[12] Galbraith-Emami S., Lobstein T. (2013). The impact of initiatives to limit the advertising of food and beverage products to children: A systematic review. Obes. Rev..

[13] Watson W.L., Lau V., Wellard L., Hughes C., Chapman K. (2017). Advertising to children initiatives have not reduced unhealthy food advertising on Australian television. J. Public Health.

[14] The Australian Communications and Media Authority (2017). Children’s Television Viewing and Multi-Screen Behaviour.

[15] Smithers L.G., Lynch J.W., Merlin T. (2012). Television Marketing of Unhealthy Food and Beverages to Children in Australia: A Review of Published Evidence from 2009.

[16] Cairns G., Angus K., Hastings G., Caraher M. (2013). Systematic reviews of the evidence on the nature, extent and effects of food marketing to children. A retrospective summary. Appetite.

[17] Dhar T., Baylis K. (2011). Fast-food consumption and the ban on advertising targeting children: The Quebec experience. J. Mark. Res..

[18] Silva A., Higgins L.M., Hussein M. (2015). An Evaluation of the Effect of Child-Directed Television Food Advertising Regulation in the United Kingdom. Can. J. Agric. Econ. Revue Can. D’agroecon..

[19] Veerman J.L., Van Beeck E.F., Barendregt J.J., Mackenbach J.P. (2009). By how much would limiting TV food advertising reduce childhood obesity?. Eur. J. Public Health.

[20] Goris J.M., Petersen S., Stamatakis E., Veerman J.L. (2010). Television food advertising and the prevalence of childhood overweight and obesity: A multicountry comparison. Public Health Nutr..

[21] Magnus A., Haby M., Carter R., Swinburn B. (2009). The cost-effectiveness of removing television advertising of high-fat and/or high-sugar food and beverages to Australian children. Int. J. Obes..

[22] Cecchini M., Sassi F., Lauer J.A., Lee Y.Y., Guajardo-Barron V., Chisholm D. (2010). Tackling of unhealthy diets, physical inactivity, and obesity: Health effects and cost-effectiveness. Lancet.

[23] Sonneville K.R., Long M.W., Ward Z.J., Resch S.C., Wang Y.C., Pomeranz J.L., Moodie M.L., Carter R., Sacks G., Swinburn B.A. (2015). BMI and healthcare cost impact of eliminating tax subsidy for advertising unhealthy food to youth. Am. J. Prev. Med..

[24] Chou S.-Y., Rashad I., Grossman M. (2008). Fast-food restaurant advertising on television and its influence on childhood obesity. J. Law Econ..

[25] Robinson T.N. (1999). Reducing children’s television viewing to prevent obesity: A randomized controlled trial. JAMA.

[26] Gorn G.J., Goldberg M.E. (1982). Behavioral evidence of the effects of televised food messages on children. J. Consum. Res..

[27] Bolton R.N. (1983). Modeling the impact of television food advertising on children’s diets. Curr. Issues Res. Advert..

[28] Obesity Policy Coalition and the Global Obesity Centre (2017). Tipping the Scales, Australian Obesity Prevention Consensus.

[29] The Australian Prevention Partnership Centre Deakin University and Informas (2017). Policies for Tackling Obesity and Creating Healthier Food Environments.

[30] Australian Communications and Media Authority (2015). Children’s Television Viewing, Research Overview.

[31] Boyland E.J., Whalen R. (2015). Food advertising to children and its effects on diet: Review of recent prevalence and impact data. Pediatr. Diabetes.

[32] Sadeghirad B., Duhaney T., Motaghipisheh S., Campbell N., Johnston B. (2016). Influence of unhealthy food and beverage marketing on children’s dietary intake and preference: A systematic review and meta-analysis of randomized trials. Obes. Rev..

[33] Boyland E.J., Nolan S., Kelly B., Tudur-Smith C., Jones A., Halford J.C., Robinson E. (2016). Advertising as a cue to consume: A systematic review and meta-analysis of the effects of acute exposure to unhealthy food and nonalcoholic beverage advertising on intake in children and adults. Am. J. Clin. Nutr..

[34] Lusk J.L., Roosen J., Shogren J.F. (2011). The Oxford Handbook of the Economics of Food Consumption and Policy.

[35] Cecil J.E., Palmer C.N., Wrieden W., Murrie I., Bolton-Smith C., Watt P., Wallis D.J., Hetherington M.M. (2005). Energy intakes of children after preloads: Adjustment, not compensation. Am. J. Clin. Nutr..

[36] Hall K.D., Butte N.F., Swinburn B.A., Chow C.C. (2013). Dynamics of childhood growth and obesity: Development and validation of a quantitative mathematical model. Lancet Diabetes Endocrinol..

[37] Lal A., Mantilla-Herrera A.M., Veerman L., Backholer K., Sacks G., Moodie M., Siahpush M., Carter R., Peeters A. (2017). Modelled health benefits of a sugar-sweetened beverage tax across different socioeconomic groups in Australia: A cost-effectiveness and equity analysis. PLoS Med..

[38] Australian Bureau of Statistics (2010). 6302.0-Average Weekly Earnings, Australia, May 2010.

[39] Australian Bureau of Statistics (2012). 6348.0-Labour Costs, Australia, 2010-11.

[40] Fair Work Ombudsman (2017). Payment for Annual Leave.

[41] Australian Institute of Health and Welfare (2004). Health System Expenditure on Disease and Injury in Australia, 2000-01.

[42] Australian Institute of Health and Welfare (2014). Health Expenditure 2012-13.

[43] Australian Bureau of Statistics (2017). Socio-Economic Indexes for Areas.

[44] Australian Bureau of Statistics (2013). 4324.0.55.002-Microdata: Australian Health Survey: Nutrition and Physical Activity, 2011-12.

[45] Murray C.J., Abraham J., Ali M.K., Alvarado M., Atkinson C., Baddour L.M., Bartels D.H., Benjamin E.J., Bhalla K., Birbeck G. (2013). The state of US health, 1990–2010: Burden of diseases, injuries, and risk factors. JAMA.

[46] Crino M., Herrera A.M.M., Ananthapavan J., Wu J.H., Neal B., Lee Y.Y., Zheng M., Lal A., Sacks G. (2017). Modelled Cost-Effectiveness of a Package Size Cap and a Kilojoule Reduction Intervention to Reduce Energy Intake from Sugar-Sweetened Beverages in Australia. Nutrients.

[47] EpiGear International (2016). Ersatz.

[48] Chen G., Ratcliffe J., Olds T., Magarey A., Jones M., Leslie E. (2014). BMI, health behaviors, and quality of life in children and adolescents: A school-based study. Pediatrics.

[49] George B., Harris A., Mitchell A. (2001). Cost-effectiveness analysis and the consistency of decision making. Pharmacoeconomics.

[50] OzTAM (2016). Australian Multi-Screen Report.

[51] FreeTV Australia (2010). Advertising Revenue for Commercial Television Networks, January to June 2010. http://www.freetv.com.au/media/News-Media_Release/PR14_Advertising_revenue_for_commercial_television_networks_-_Jan-Jun_2010.pdf.

[52] FreeTV Australia (2011). Advertising Revenue for Commercial Television Networks, July to December 2010. http://www.freetv.com.au/Media/News-Media_Release/Revenue_figures_July_Dec_2010.pdf.

[53] Adnews (2016). Where’s the Money Going? Exclusive ad Spend Trends Report.

[54] Deloitte Australia (2016). Media Consumer Survey 2016, Australian Media and Digital Preferences.

[55] Vos T., Carter R., Barendregt J., Mihalopoulos C., Veerman L., Magnus A. (2010). Assessing Cost-Effectiveness in Prevention.

[56] Parents’ Voice Junk Food Marketing 2017. https://parentsvoice.org.au/our-work/junk-food-marketing/.

[57] Australian Communications and Media Authority (2007). Children’s Television Standards Review.

[58] Mialon M., Swinburn B., Allender S., Sacks G. (2016). Systematic examination of publicly-available information reveals the diverse and extensive corporate political activity of the food industry in Australia. BMC Public Health.

[59] Brown V., Moodie M., Cobiac L., Herrera A.M., Carter R. (2017). Obesity-related health impacts of fuel excise taxation-an evidence review and cost-effectiveness study. BMC Public Health.

