# Supplementary material for: The Potential Cost-Effectiveness and Equity Impacts of Restricting Television Advertising of Unhealthy Food and Beverages to Australian Children
Source: Nutrients. 2018 May 15;10(5):622. doi: 10.3390/nu10050622 (PMC5986502; doi:10.3390/nu10050622)
Supplement: Supplementary file 1 [file nutrients-10-00622-s001.pdf]

Supplementary information 1. Summary of Published Findings on the Health Benefits of Restricting TV Advertising of HFSS Food and Beverages to Children

Table S1.1 Summary of published findings on the health benefits of restricting TV advertising of HFSS food and beverages to children.

Study	Population	Evidence of Effect Derived From	Mean BMI Effect	Time Horizon	Health Benefits (95% UI)	Healthcare Cost-Savings (95% UI)	ICER
Cecchini et al. [22]	2–18 year olds, 7 countries	Chou et al. [24]<br>Cross-sectional<br>Fast-food restaurant TV ads association with BMI	−0.03 to −0.78 kg/m^2	20 years or 50 years	20 years: Range from 38 DALYs (Brazil) to 288 DALYs (Russia)<br>50 years: Range from 610 DALYs (South Africa) to 5 823 DALYs (Russia)	N/A	Cost-effective, some Dominant *
Goris et al. [20]	6–11 year olds, 6 countries	Bolton et al. [27]<br>Cross-sectional<br>Effect of TV ad exposure on energy intake<br>Delphi study	−0.38 kg/m^2<br>−1 kg/m^2	N/A	The contribution of TV food advertising to prevalence of obesity is between 4–40% (dependent on country setting, effect size)	N/A	N/A
Sonneville et al. [23]	2–19 year olds, USA	Robinson et al. [25]<br>RCT<br>Effect of reduced TV time on body mass index	−0.028 kg/m^2	Effect: 2 years<br>Outcomes: 10 years	4 538 QALYs (1752–7489)	USD352M<br>(USD138M–581M)	Dominant *
Magnus et al. [21]	5–14 year olds, Australia	Gorn & Goldberg [26]<br>RCT<br>Effect of TV ad exposure on energy intake	Food: −0.13 kg/m^2 (95% UI −0.03, −0.25)<br>Beverages: −0.04 kg/m^2 (95% UI −0.01, −0.08)	Lifetime	37 000 DALYs<br>(16 000–59 000)	AUD300M<br>(AUD130M–480M)	Dominant *
Veerman et al. [19]	6–12 year olds, USA	Bolton et al. [27]<br>Cross-sectional<br>Effect of TV ad exposure on energy intake<br>Delphi study	−0.38 kg/m^2<br>−1 kg/m^2	N/A	Decrease in the prevalence of obesity by 2.7% (95% UI 2.3–3.1%) boys, 2.4% (95% UI 2.1–2.8%) girls<br>Decrease in the prevalence of obesity by 6.8% (95% UI 3.9–10.1%) boys, 6% (95% UI 3.5–8.7%) girls	N/A	N/A
Our findings	5–15 year olds	Meta-analysis<br>Effect of TV ad exposure on energy intake	−0.352 kg/m^2	Lifetime	88 396<br>(95% UI 54 559–123 199)	AUD783.8M<br>(95% UI AUD375.6M–1.2B)	Dominant *

* Dominant intervention results in health gains and cost-savings; 95% UI = 95% uncertainty interval; Assoc = association; AUD = Australian dollars; BMI = body mass index, measured as weight in kilograms divided by height in metres squared; DALY = disability adjusted life year; HALY = health adjusted life year; ICER =

incremental cost-effectiveness ratio; Kg = kilogram; QALY = quality adjusted life year; M = million; m = metres; USA = United States of America; USD = United States dollars.

Supplementary information 2. Scoping Search Strategy

Table S2.1 Scoping search strategy.

Search Identifier	Key Words
1	weight OR overweight OR obes * OR "body mass index" OR BMI
2	advertis* OR marketing OR television OR TV
3	child* OR adolescen * OR youth
4	"random * control * trial" OR RCT OR experiment*.
5	Consum * OR food OR "energy intake"
6	"systematic review" OR review OR "meta analysis" OR "meta-analysis"

1 AND 2

1 AND 2 AND 3

1 AND 2 AND 3 AND 4

2 AND 4

2 AND 3

2 AND 3 AND 4

2 AND 3 AND 5 AND 6

Supplementary information 3. Estimate of Effect, Meta-Analysis Results

A meta-analysis of relevant studies reported in Boyland et al. [33] was undertaken. Studies were selected for inclusion into our meta-analysis if they were conducted in children and reported exposure and an effect expressed as a change in kilocalories between intervention and control. Study characteristics of the included studies into our meta-analysis are given in Table A3.

Table S3.1. Characteristics of included studies in the meta-analysis. .

Study	Study Type	Aim	Population	N	Ad Exposure	Ad Conditions	Outcome
Dovey et al. 2011 [60]	E, WS	To explore the role of food neophobia in responsiveness to food adverts in children	UK children aged 5–7 years	66	2 min ads in 14 min cartoon	(i) healthy foods (ii) unhealthy foods (iii) toys	Food intake (kcal). Food items offered: chocolate, jelly sweets, potato crisps, Snack-a-jacks, grapes, carrot sticks
Halford et al. 2007 [61]	E, WS	To explore the effects of food advert exposure on young children	UK children aged 5–7 years	93	10 ads in a 10 min cartoon*	(i) food-related adverts (ii) non-food related adverts (iii) a cartoon	Food intake (kcal). Food items offered: chocolate, jelly sweets, potato crisps, Snack-a-jacks, grapes
Halford et al. 2008 [62]	E, WS	To explore the effect of food advert exposure on children's food intake	UK children aged 9–11 years	59	10 ads in a 10 min cartoon. Ads approx. 30 s in length	(i) food-related adverts (ii) non-food related adverts (iii) a cartoon	Food intake (kcal). Food items offered: chocolate, jelly sweets, potato crisps, Snack-a-jacks, grapes

Ads = TV advertisements; E = experiment; Kcal = kilocalories; min = minutes; UK = United Kingdom; WS = within subject experimental design; * Ad length assumed 30 s.

The inverse variance method was used, assuming a random effects model (Table A4). Tests for heterogeneity were performed using I^2 and Cochran's Q test. Heterogeneity was regarded as substantial when I^2 exceeded 40% or the Q statistic was significant at $p < 0.10$. Potential publication and small study bias was examined visually for the primary meta-analysis using funnel and Doi plots, where a symmetrical plot suggests no or little bias (Figure A1). The Luis Furuya-Kanamori (LFK) index of asymmetry is also presented from the Doi plot for the

primary meta-analysis, with an assessment of "no", "minor" or "major" asymmetry. Sensitivity analyses were conducted by omitting individual studies (Table A4). Sensitivity analysis 1 values displayed less heterogeneity, and so was used as the input parameter to the worst case sensitivity analysis.

Table S3.2. Results of meta-analysis, effect estimate for use in scenario analyses. kcal/min = kilocalories per minute.

	Primary Meta-Analysis	(SA1) Omit Halford et al. 2008	(SA2) Omit Halford et al. 2007	(SA3) Omit Dovey et al. 2011
**Included studies in sub-analysis**	Dovey et al. 2011<br>Halford et al. 2007<br>Halford et al. 2008	Dovey et al. 2011<br>Halford et al. 2007	Dovey et al. 2011<br>Halford et al. 2008	Halford et al. 2007<br>Halford et al. 2008
**Pooled estimate weighted mean difference kcal/min exposed, RE**	37.94 (95% UI 15.57–60.32)	27.6 (95% UI 19.5–35.7)	45.8 (95% UI 15.2–76.4)	41.8 (95% UI 2.4–81.2)
**Cochran's Q**	23.2 (p = 0.00)	1.15 (p = 0.283)	16.9 (p = 0.00)	18.7 (p = 0.00)
**I^2**	91.4 (95% UI 77.8–96.7)	13.2 (95% UI 0–0)	94.1 (95% UI 81.3–98.1)	94.6 (95% UI 83.6–98.2)
**LFK Index**	0.89 (No asymmetry)	-	-	-

LFK Index = Luis Furuya-Kanamori Index; RE = random effects; SA = sensitivity analysis; 95% UI = 95% uncertainty interval.

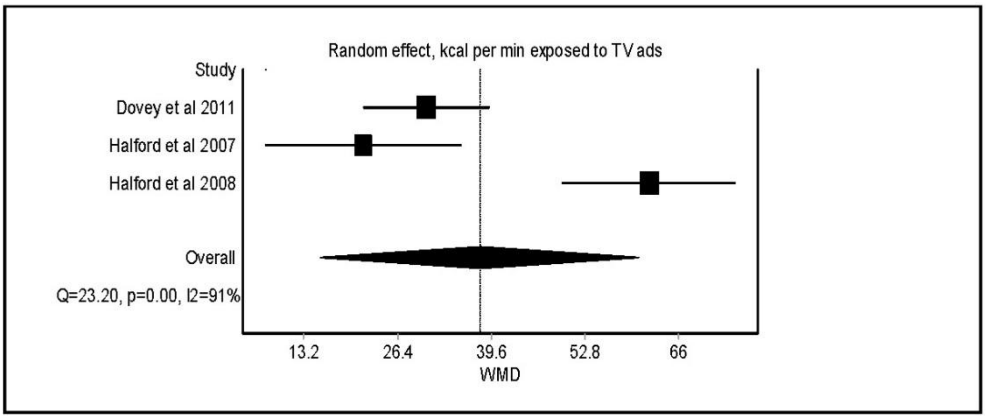

Figure S3.1. Forest plot of meta-analysis, primary analysis effect estimate for use in Scenario 2. Kcal = kilocalories; min = minute; TV ads = television advertisements; WMD = weighted mean difference.

Supplementary information 4. Mean Minutes Spent Watching TV per Day, by Age and Quintile

Table S4.1. Mean minutes spent watching TV per day, by age and quintile.

AGE	Q1	Q5	ALL
**5**	70.6<br>(95% UI 53.9–87.1)	59.9<br>(95% UI 45.1–75.5)	70.8<br>(95% UI 63.1–78.7)
**6**	92.6<br>(95% UI 69.5–115.2)	76.3<br>(95% UI 57.6–94.8)	77.2<br>(95% UI 65.9–88.2)
**7**	73.3<br>(95% UI 47.6–98.8)	71<br>(95% UI 52.4.6–89.5)	75<br>(95% UI 64.5–85.5)
**8**	74<br>(95% UI 42.7–106.7)	70<br>(95% UI 44–95.2)	78.2<br>(95% UI 68.2–88.2)
**9**	85.6<br>(95% UI 58.9–111.8)	59<br>(95% UI 37.4–80.2)	76.7<br>(95% UI 65.2–88.4)
**10**	113.4<br>(95% UI 66.6–160.6)	62.1<br>(95% UI 41.2–82.4)	81.6<br>(95% UI 70.4–92.5)
**11**	105.2<br>(95% UI 76.6–133.3)	70.2<br>(95% UI 48.5–92.4)	89.6<br>(95% UI 74.8–103.6)
**12**	140.5<br>(95% UI 77.2–202.7)	65.2<br>(95% UI 41.6–88.5)	97.2<br>(95% UI 80.9–114.4)
**13**	90.8	72.5	80.6

	(95% UI 3.7–177.5)	(95% UI 52.5–94.2)	(95% UI 68.1–93.3)
**14**	87.6<br>(95% UI 33–142)	80.9<br>(95% UI 34.5–125.6)	85.5<br>(95% UI 71–100.2)
**15**	69.2<br>(95% UI 39.8–97.9)	62.4<br>(95% UI 38.6–85.9)	76<br>(95% UI 63–88.9)

Source: Australian Health Survey 2011–2012 [44].

Supplementary information 5. Sensitivity Analysis Results

Table S5.1. One-way sensitivity analysis, assumed loss of network revenue.

Results	Children (5–17 Years)	Children Q1<br>(Most Disadvantaged)	Children Q5<br>(Least Disadvantaged)
**One-way sensitivity analysis:** *Assumed loss of network revenue, year one of intervention*			
Mean modelled kJ effect per day, children aged five to 15 years	−115 kJ/day	−132 kJ/day	−97 kJ/day
Mean modelled BMI effect, children aged five to 15 years	−0.352 kg/m^2	−0.395 kg/m^2	−0.299 kg/m^2
Mean BMI effect maintained in adulthood	−0.345 kg/m^2	−0.313 kg/m^2	−0.282 kg/m^2
Total HALYS saved over lifetime	88 453<br>(95% UI 53 764–123 373)	17 270<br>(95% UI 10 323–24 572)	11 265<br>(95% UI 6 878–15 642)
Total healthcare cost-savings over lifetime	AUD787.8M<br>(95% UI AUD372.8M–1.2B)	AUD125.2M<br>(95% UI AUD60.3M–189.1M)	AUD91.3M<br>(95% UI AUD47.4M–138.9M)
Total intervention costs	AUD105.4M<br>(95% UI AUD105.3M–105.4M)	AUD21.2M<br>(95% UI AUD17.7M–25.8M)	AUD21.2M<br>(95% UI AUD16.7M–27M)
Total net cost	AUD682.5M<br>(95% UI AUD267.4M–1.1B)	AUD104M<br>(95% UI AUD34.5M–171.4M)	AUD70.3M<br>(95% UI AUD20.4M–122.2M)
Net cost per HALY saved (ICER)	Dominant *	Dominant *	Dominant *
Probability of dominance	99.9%	100%	99.7%
Probability of cost-effectiveness	100%	100%	100%

95% UI = 95% uncertainty interval based on 2000 simulations; AUD = Australian dollars; BMI = body mass index; HALYs = Health adjusted life years; ICER = Incremental cost-effectiveness ratio; kJ = kilojoule. 1 kilocalorie is equal to 4.184 kilojoules; Q = SEIFA IRSD quintile; * Dominant interventions result in health gains and cost-savings.

Table S5.2 Worst-case multi-variate sensitivity analyses.

Results	Children (5–17 Years)	Children Q1<br>(Most Disadvantaged)	Children Q5<br>(Least Disadvantaged)
Worst-case multi-variate sensitivity analysis:<br>• Assumed loss of network revenue, year one of intervention<br>• Pooled WMD kcal per minute exposed to TV ads (WMD 27.6, 95% CI 19.5–35.7)<br>• Adjustment factor for application of experimental effect to real-world setting (sampled from a Pert distribution, minimum 0%, most likely 75%, maximum 100%)<br>• Adjustment factor for proportion of daily time spent watching TV via paid or streamed services (assuming no advertising content) (sampled from a Pert distribution, minimum 20%, most likely 22%, maximum 24%)			
Mean modelled kJ effect per day, children aged five to 15 years	−43 kJ/day	−48 kJ/day	−36 kJ/day
Mean modelled BMI effect, children aged five to 15 years	−0.13 kg/m^2	−0.15 kg/m2	−0.11 kg/m2
Mean BMI effect maintained in adulthood	−0.13 kg/m^2	−0.12 kg/m^2	−0.11 kg/m^2
Total HALYS saved over lifetime	33 463<br>(95% UI 4 299–89 269)	6 595<br>(95% UI 914–18 740)	4 375<br>(95% UI 482–12 705)
Total healthcare cost-savings over lifetime	AUD295.9M<br>(95% UI AUD33.8M–815.9M)	AUD47.9M<br>(95% UI AUD6.3M–143.9M)	AUD35.4M<br>(95% UI AUD3.5M–107.1M)
Total intervention costs	AUD104.6M<br>(95% UI AUD83.8M–132.4M)	AUD21.1M<br>(95% UI AUD21.1M–21.2M)	AUD21.1M<br>(95% UI AU21.1M–21.2M)
Total net cost	AUD191.3M<br>(95% UI –AUD732M–98.5M)	AUD26.8M<br>(95% UI -AUD122.7M–14.8M)	AUD14.4M<br>(95% UI –AUD86.1M–17.6M)
Net cost per HALY saved (ICER)	Dominant *<br>(95% UI dominant-AUD16 463)	Dominant *<br>(95% UI dominant-AUD16 342)	Dominant *<br>(95% UI dominant-AUD35 819)
Probability of dominance	83.5%	77.7%	62.7%
Probability of cost-effectiveness	99.5%	99.7%	98.4%

95% UI = 95% uncertainty interval based on 2000 simulations; AUD = Australian dollars; BMI = body mass index; HALYs = Health adjusted life years; ICER = Incremental cost-effectiveness ratio; kJ =

kilojoule. 1 kilocalorie is equal to 4.184 kilojoules; Q = SEIFA IRSD quintile; * Dominant interventions result in health gains and cost-savings.

References

60. Dovey, T.M.; Taylor, L.; Stow, R.; Boyland, E.J.; Halford, J.C. Responsiveness to healthy television (TV) food advertisements/commercials is only evident in children under the age of seven with low food neophobia. *Appetite* **2011**, *56*, 440–446.
61. Halford, J.C.; Boyland, E.J.; Hughes, G.; Oliveira, L.P.; Dovey, T.M. Beyond-brand effect of television (TV) food advertisements/commercials on caloric intake and food choice of 5–7-year-old children. *Appetite* **2007**, *49*, 263–267.
62. Halford, J.C.; Boyland, E.J.; Hughes, G.M.; Stacey, L.; McKean, S.; Dovey, T.M. Beyond-brand effect of television food advertisements on food choice in children: The effects of weight status. *Public Health Nutr.* **2008**, *11*, 897–904.
